# Uric Acid as a Cause of Asthma

**Published:** 1899-02

**Authors:** L. H. Watson

**Affiliations:** Chicago, Ill.; 100 State street


### URIC ACID AS A CAUSE OF ASTHMA.

BY L. H. WATSON, M.D., CHICAGO, ILL.

Asthma is usually considered as a neurotic affection, dependent upon some irritation of the pneumogastric nerve, and characterized by bronchial spasm, and accompanied by an exudate of mucin.

While this may be a fair statement of the condition, it does not explain the etiology of the disease. An irritable condition of the respiratory nerve centers, caused by an impression made upon them, either through direct contact, or through morbid material in the blood current, exciting a reflex spasm, would seem to be the principal agent in producing this paroxysmal condition called asthma. Long have we sought the cause and found it not. Von Leyden thought he had it, when he exploited the Charcot-Leyden crystals and Cusschmann's spirals; now we know they are only an accompaniment, and while diagnostic of asthma, do not at all account for its existence. They are simply confirmatory signs when the diagnosis is complicated; indeed the crystals are said to be found only late in the attack. We have been accustomed to divide asthma into two classes, idiopathic and secondary, but it is extremely doubtful if we see a case of genuine idiopathic asthma. By searching we can trace a cause immediate or remote. We have mechanical causes, like thymic asthma, nasal polypi, the pollen of plants, drug dust, disease of the spine or heart. We have chemical causes of a toxic nature, due to blood toxins taken up from the stomach or intestines, and failing of elimination by emunctories, remain as elements of discord exciting the paroxysms.

Heredity has been supposed to play a large part in attacks, but only through hypersensitiveness of the nervous system. Locality undoubtedly influences the recurrence of the spasm. Some people can not exist comfortably in the country, and are free from attacks in town. I once had a patient, a very intelligent man, who could not sleep on the ground floor of his house but was free of attacks in the second story. Many idiosyncracies attach themselves to attacks of asthma, and it would require a larger article than this to trace them all.

I desire to call attention particularly to the "uric acid storm" as a factor, and a large one, in the production of the asthmatic paroxysms. Haig says in *International Clinics*, Vol. III:

"My researches leave little doubt that asthma represents the effect of uric acid on the circulation in the thorax, and that it is paroxysmal for the same reason that epilepsy and migrain are paroxysmal, in accordance with the natural fluctuation of the uric acid, and the amount of that substance passing through the blood, and furthermore the only way to treat asthma is to clean the blood of uric acid and keep it clean."

This is a strong statement, but facts bear out Haig's assertion. Long before the uric acid hypothesis was understood, physicians depended upon iodide of potash as a curative remedy, but it was not found of great service in dispelling the attacks. The use for two or three weeks before an expected paroxysm seemed to abort it, and the iodide was deemed almost a specific. What it did do was to cause the elimination of uric acid and thus lessen the irritation upon the vagi nerves, which precipitated this attack. It remained for Haig to explain the reason in an intelligent and scientific manner.

While I will not go so far as to say that all cases of asthma are caused by uric acid, I do say that almost all cases are benefited by attention to the elimination of uric acid, and many cases are absolutely cured when the proper methods are adoped and certain dietary plans are accepted which shall prevent its accumulation It is a well-known fact, well-known especially to asthmatics, that they can not transgress the rules which govern proper digestion and assimilation, or they will pay the penalty.

My first method is to insist upon a rigid diet list, excluding all the producers of nitrogen, and then begin the treatment with thialion, which is certainly one of the most efficacious remedies we now possess for removing the excess of uric acid in the blood and picking up the deposits in the forms of urates which have been already deposited to the tissues, ready to contaminate the blood stream when the conditions are favorable. Abjure them all nitrogenous supplies, and put the patient upon thialion for two or three weeks; longer if the case be an obstinate one. You will be fully repaid for the attempt.

There is no doubt also that uric acidæmia when it contracts the arteriols will certainly suspend gastro-intestinal digestion and absorption, and allow putrefactive processes to take place, which shall furnish toxins that will find their way into the circulation, and thus again act as irritants, while producing high arterial tension. An asthmatic attack represents the thoracic effect of this tension. Two confirmatory facts would seem to favor Haig's hypothesis; the first is that most attacks of asthma occur at from 2 to 4 o'clock in the morning, when the uric acid flood is at its height, and the other is that after an attack of asthma, as after a uric acid storm, there is a flow of limpid, pale urine in great abundance. Upon the whole the uric acid theory offers us, perhaps, the most feasible theory known at the present day to account for the peculiarity of asthmatic attacks. I append two typical cases.

Miss L—, a maiden lady, 50 years of age, a long sufferer from hay fever, which usually begins in August and lasts until the first frost. In November, 1898, she suffered from persistent asthmatic attacks which were supposed to be due to the hay fever. Obtaining only small relief from all the usual remedies she placed herself under the care of a specialist, who proceeded to cauterize and burn out the redundant nasal mucosæ, which seemed to be the irritating cause of her attacks. The asthma continuing, she came under my care. Discovering her to be a confirmed dyspeptic, I first attended to her diet, and placed her upon thialion. In a couple of weeks relief came and in six weeks after the treatment was commenced, she had no further attack.

The second case was that of an old asthmatic, Mr. K—, who was also an old dyspeptic. Winter and summer this gentleman, who possessed a large amount of this world's goods, was constantly using Himrod's pastiles and cursing his fate. Thialion combined with treatment directed to get his stomach in fair condition, has so relieved him that I can not persuade him to stop its use. He takes it constantly every morning in hot water, and while he wheezes a little now and then when he has been indiscreet at table, he is practically well.

100 State street.

---

THE TREATMENT OF GASTRIC ULCER.—By Dr. Wm. Murrell (*The Therapist*, Sept. 15, 1898).—After following out the orthodox and generally accepted treatment, he prescribes five drops of tincture of iodin (U. S. P.) three times a day in a wineglass of water. It produces no pain or inconvenience, and the condition of the gastric mucosa rapidly improves until, in a few days, the ulcer is completely healed and the digestive powers are restored to their pristine condition. Iodism has never resulted in any case as yet. In the early stages it will often effect a cure without limitation of diet or confinement in bed. Should the patient object to the pungency of the tincture of iodin, add half a drachm of glycerin to each dose.

Prof. T. R. Fraser, of Edinburgh, has adopted a modification of this treatment. He gives chromic acid, or, to speak more correctly, bichromate of potash, one-twelfth of a grain three times a day after active digestion has taken place. It is usually given in pill form, or simple solution in water flavored with orange.

